# Using SEM-EDX and ICP-OES to Investigate the Elemental Composition of Green Macroalga *Vaucheria sessilis*

**DOI:** 10.1155/2014/891928

**Published:** 2014-08-11

**Authors:** Izabela Michalak, Krzysztof Marycz, Katarzyna Basińska, Katarzyna Chojnacka

**Affiliations:** ^1^Department of Chemistry, Institute of Inorganic Technology and Mineral Fertilizers, Wrocław University of Technology, Smoluchowskiego 25, 50-372 Wrocław, Poland; ^2^Department of Environment Hygiene and Animal Welfare, Electron Microscope Laboratory, Environmental and Life Sciences University, Chełmońskiego 38c, 50-630 Wrocław, Poland

## Abstract

The biomass of *Vaucheria sessilis* forms algal mats in many freshwaters. There is a need to find the method of algal biomass utilization. *Vaucheria sessilis* is a rich source of micro- and macronutrients and can be used as a soil amendment. In the paper, the elemental composition of enriched, via bioaccumulation process, macroalga was investigated. For this purpose, two independent techniques were used: scanning electron microscopy with an energy dispersive X-ray analytical system (SEMEDX) and inductively coupled plasma optical emission spectroscopy (ICP-OES). The biomass was exposed to two microelemental solutions, with Cu(II) and Zn(II) ions. After two weeks of the experiment, macroalga accumulated 98.5 mg of Zn(II) ions in 1 g of dry biomass and 68.9 mg g^−1^ of Cu(II) ions. Micrographs performed by SEM proved that bioaccumulation occurred. Metal ions were bound on the surface and in the interior of cells. Mappings of all cations showed that in the case of the surface of biomass (biosorption), the elements constituted aggregations and in the case of the cross section (bioaccumulation) they were evenly distributed. The algal biomass with permanently bound microelements can find an application in many branches of the industry (feed, natural fertilizers, etc.).

## 1. Introduction

Algae can be used as fertilizers, soil conditioners, and biostimulants and are a source of livestock feed [1]. The interest in this category of bioproducts in modern agriculture results from the trend to search for new preparations based on natural substances that replace (eliminate) the application of chemicals in agriculture. Macroalgae can be applied on soils, in hydroponic solutions, or in the form of foliar applications for plants [2–5]. The multiplicity of applications of algal biomass results from their natural properties. Macroalgae contain a large number of organic and mineral compounds (micro- and macronutrients). They are particularly rich in phytohormones (indoleacetic acids (IAA), commonly known as auxins, gibberellic acids, cytokinins, abscisic acids (ABA), and ethylene), complex organic compounds, vitamins, simple and complex sugars (polysaccharides like alginates, laminarian, and carrageenans), enzymes, N-containing compounds like betaines, proteins, and amino acids, sterols [4, 6].

Additionally, the biomass possesses a high ability to bioaccumulate metal ions from the aqueous solution. This causes the biomass to also be able to serve as a mineral feed supplement for livestock [7, 8] and bioaccumulator in biological wastewater treatment processes and in bioremediation technologies [9, 10] or as a bioindicator, since it provides a time-integrated picture of the bioavailable pollutants [11].

The ability of the biomass to bind metal ions is strongly dependent on the surface. The surface of the biomass was widely examined for the presence of functional groups with the use of various analytical techniques such as titration, XPS, FTIR, and SEM-EDX [12]. Some of the active groups (carboxylates, sulfhydryls, phosphates, sulfates, and hydroxyls) present on the surface are negatively charged and able to bind metal cations while amine and imidazole groups (positively charged) can bind negatively charged metal complexes [13].

The aim of the current paper was to evaluate the bioaccumulation properties of macroalga, to present how metal ions were accumulated on the surface and in the cross section of the biomass, and to find the potential application for the biomass enriched with metal ions. Therefore, two methods were chosen: scanning electron microscopy with an energy dispersive X-ray analytical system (SEM-EDX) and inductively coupled plasma optical emission spectroscopy (ICP-OES). Scanning electron microscopy is a powerful technique which can be used to investigate the binding of metal ions to seaweed. SEM allows evaluating morphological changes on the surface, for example, changes in the cell wall composition after the metal ions binding. When SEM is combined with EDX technique, it can provide valuable input in determining the distribution of various elements on the seaweed surface [14]. It should be emphasized that SEM provides only a qualitative estimation of the surface structure.

To obtain quantitative information regarding changes in elemental composition of the used biomass and/or solution, an additional technique is required. For solutions where concentration of many elements is very low highly sensitive methods should be used—for example, inductively coupled plasma optical emission spectroscopy (ICP-OES). Varian Vista-MPX equipment used in the present work offers a host of benefits, which include the productivity of simultaneous measurement of all elements from parts-per-billion to percent levels, simple “one step” analysis, fast optimization, and hands-free operation, from full PC control of all instrument parameters [15].

In literature, both methods, SEM-EDX and ICP-OES, are used to investigate the bioaccumulation of metal ions by biomass, but the data is insufficient and general. It is indicated that changes in surface morphology are usually related to disruption of the cross-linking between the metal ion and negatively charged chemical groups, for example, carboxyl groups in the cell wall polymers. Raw seaweed usually contains high contents of calcium and magnesium (naturally present from seawater) in the cell wall creating a net of cross-linking [16]. When the seaweed is exposed to metal ions solutions, for example, cadmium, these cations replace some of the calcium and magnesium ions thus changing the nature of the cross-linking on the surface and resulting in morphological changes. Yang and Chen [17] observed surface protuberance and microstructures in the raw seaweed of* Sargassum* sp. after biosorption of hexavalent chromium and suggested that this may be due to calcium and another salt crystalloid deposition. This was in agreement with their EDX analysis which showed that calcium was a major component of the seaweed surface.

In the current paper, bioaccumulation properties of macroalga* Vaucheria sessilis* (*Xanthophyceae*) towards Cu(II) and Zn(II) ions are presented. Additionally, the morphological changes of the surface of the biomass after bioaccumulation and the arrangement of metal cations on the surface and in the cross section of the biomass were investigated. Permanently bound microelement ions with the algal biomass could constitute a highly bioavailable source of micronutrients for plants, as well as for animals.

## 2. Materials and Methods

### 2.1. Biomass of Vaucheria Sessilis

The starting culture of macroalga* Vaucheria sessilis* was obtained from the Sammlung von Algenkulturen; Albrecht-von-Haller-Institute for Plant Science; University of Göttingen and was cultivated in the laboratory according to the procedure described by Sammlung von Algenkulturen Göttingen (SAG) [18].

### 2.2. Bioaccumulation Process

Two solutions of algal medium, which contained Cu(II) and Zn(II) ions, were prepared in deionized water by dissolving appropriate amounts of CuSO_4_ *·*5H_2_O and ZnSO_4_ *·*7H_2_O (from POCh S.A. Gliwice, Poland). The concentration of each metal ion in every medium solution was 12.5 mg L^−1^. pH of the solutions was adjusted to 7 with 0.1 mol L^−1^ standardized solution NaOH/HCl (from POCh S.A. Gliwice, Poland). pH measurements were conducted with a pH-meter Mettler-Toledo—SevenMulti (Greifensee, Switzerland) equipped with an electrode InLab413 with the compensation of temperature. About 2.5 g of wet biomass of* Vaucheria sessilis* was added (about 0.14 g L^−1^ of dry biomass) into each solution. The bioaccumulation process lasted for two weeks at room temperature and daylight. After this process, the solution was filtered through filter paper. The composition of the solution was analyzed by ICP-OES, whereas the biomass was examined by SEM-EDX.

### 2.3. Analytical Methods

#### 2.3.1. Multielemental Analysis by ICP-OES

The solutions, before and after bioaccumulation process, were analysed by inductively coupled plasma optical emission spectrometer—Varian VISTA-MPX ICP-OES (Victoria, Australia) with ultrasonic nebulizer in the Chemical Laboratory of Multielemental Analyses at Wrocław University of Technology, which is accredited by ILAC-MRA and Polish Centre for Accreditation (number AB 696) according to EN-ISO 17025 [19]. For the calibration of the apparatus, the multielemental standard (100 mg L^−1^ Astasol, Czech Republic) was used. In order to prepare the calibration curve, the following working dilutions of the analytical standard were prepared: 1.0, 10, 50 mg L^−1^. As a “check standard.” the standard solution—10 mg L^−1^ was used. The acceptable result was assessed as 10%. The analytical process was controlled by the use of Certified Reference Material Hard Drinking Water (UK)—metals from LGC Standards (LGC6010) for the analysis of solutions. Values of the measurements of the CRMs were within the certified range. The examined samples were measured in three repeats. The final result was an arithmetic mean, which differed less than 5%.

#### 2.3.2. Scanning Electron Microscopy (SEM-EDX)

Natural* Vaucheria sessilis *and* Vaucheria sessilis *loaded with microelements biomass were also examined by scanning electron microscopy. The elemental analysis and mapping were performed at Wrocław University of Environmental and Life Sciences (Electron Microscope Laboratory). Samples of macroalga were fixed in 2.5% of glutaraldehyde (Sigma). Then all the samples were dehydrated by ethanol (from 30% till 100% concentration). In the next step macroalga was prepared in two planes for the observation of cross section and its surface. Samples of the macroalga were mounted on an appropriate stub and thereafter gold-sputtered (using ScanCoat six equipment—Oxford) and were observed and photographed with a scanning electron microscope—EVO LS 15 (Oberkochen, Germany), operating at 20 kV. The microscope was equipped with a BRUCKER energy dispersive X-ray system in order to obtain a distribution of elemental composition of the surface of macroalgal cell wall according to previously published data [20–22]. The X-ray spectrum of each macroalga loaded with a given microelement was obtained.

## 3. Results and Discussion

### 3.1. Multielemental Analysis of the Solution before and after Bioaccumulation Process by ICP-OES

Bioaccumulation is defined as intracellular accumulation of sorbate which occurs in two stages: the first identical with biosorption which is quick and the subsequent which is slower and includes the transport of sorbate inside the cells by an active transport system [9]. In our previous work it was shown that the rate constants for biosorption of Zn(II) and Cu(II) ions by* Vaucheria sessilis* were much higher than for bioaccumulation [7].

Table 1 presents the composition of the solution before and after bioaccumulation of Zn(II) and Cu(II) ions by* Vaucheria sessilis*. The bioaccumulation capacity was determined from the mass balance and by direct analysis. After two weeks of the experiment, the biomass of macroalga accumulated 98.5 mg of Zn(II) ions in 1 g of dry biomass and 68.9 mg g^−1^ of Cu(II) ions.

During bioaccumulation, alkali and alkaline earth metal ions were released by the biomass of* Vaucheria sessilis*. The order for both macroalgae was as follows (where MA—means macroalga): MA-Zn: K (46.6 mg g^−1^ dry mass) > Ca (22.6 mg g^−1^) > Mg (7.49 mg g^−1^) > Na (6.16 mg g^−1^) > Ba (0.129 mg g^−1^); MA-Cu: K (62.2 mg g^−1^dry mass) > Ca (32.8 mg g^−1^) > Mg (14.9 mg g^−1^) > Na (8.04 mg g^−1^) > Ba (0.433 mg g^−1^). This proves that during the first step of bioaccumulation—biosorption—alkali and alkaline earth metal ions were replaced by metal ions from the solution. It can be assumed that in the case of* Vaucheria sessilis* K(I) and Ca(II) cations played a dominating role in cation exchange in biosorption process.

### 3.2. Analysis of the Biomass after Bioaccumulation by Scanning Electron Microscopy (SEM-EDX)

SEM-EDX pictures of live macroalga* Vaucheria sessilis* were performed after two weeks of bioaccumulation of Zn(II) and Cu(II) ions. This experiment was conducted in order to prove that bioaccumulation occurred and metal ions were bound in the interior of the biomass. When the biomass is treated with metal ions, there is a possibility of transportation of these ions through the cell membrane in living cells.

**Figure 1:**
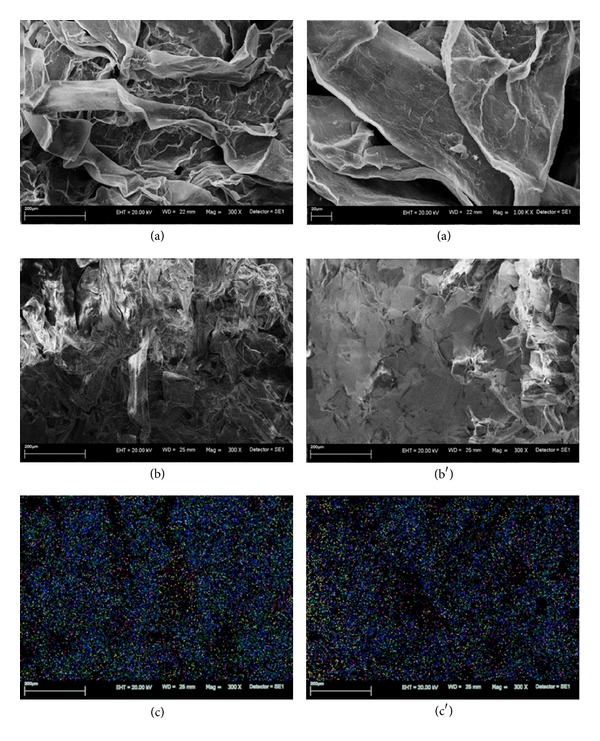
(a) A picture of the surface of natural biomass**—** *Vaucheria sessilis*; bottom picture of the surface (b) and cross section (b′) of macroalga; mapping of all elements on the surface (c) and in the cross section (c′) (C**—**green, O**—**dark blue, Na**—**bright blue, Al**—**yellow, Si**—**purple, and P—pink).

**Figure 2:**
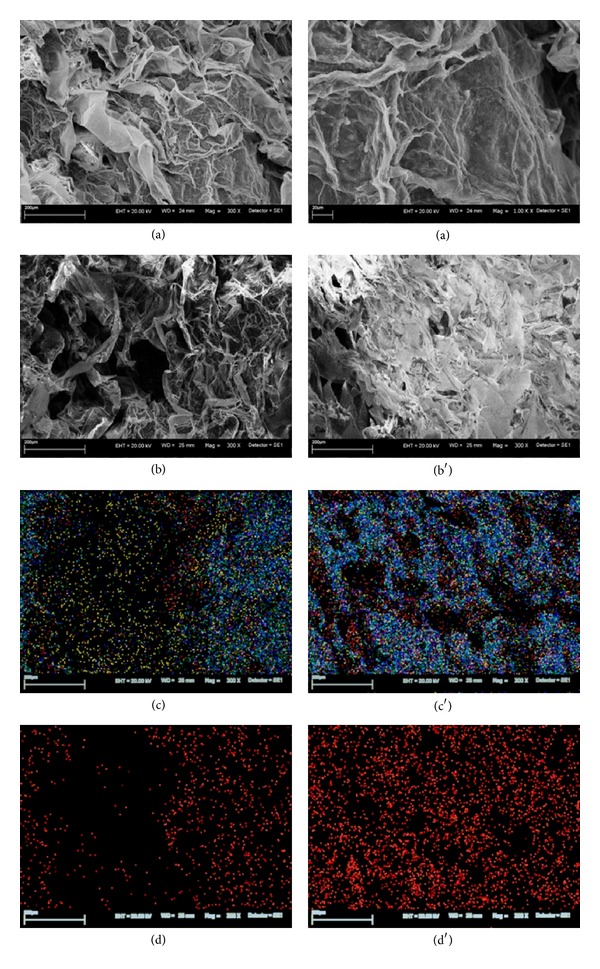
(a) A picture of the surface of* Vaucheria sessilis *enriched with Zn(II) ions; bottom picture of the surface (b) and cross section (b′) of macroalga; mapping of all elements on the surface (c) and in the cross section (c′); mapping of Zn(II) ions on the surface (d) and in the cross section (d′) (C—green, O—dark blue, Na—bright blue, Al—yellow, Zn—red, and Si—purple).

**Figure 3:**
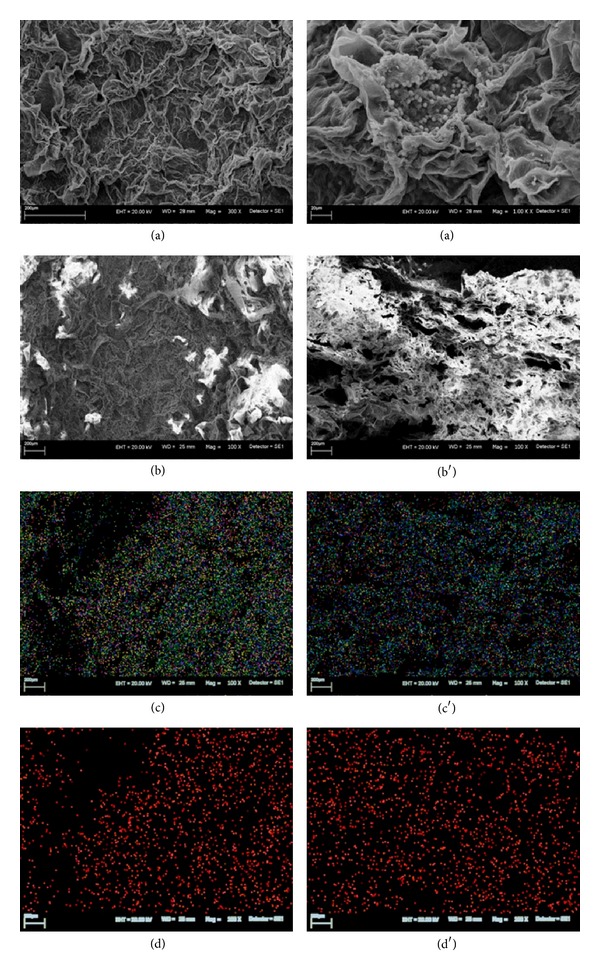
(a) A picture of the surface of* Vaucheria sessilis *enriched with Cu(II) ions; bottom picture of surface (b) and cross section (b′) of macroalga; mapping of all elements on the surface (c) and in the cross section (c′); mapping of Cu(II) ions on the surface (d) and in the cross section (d′) (C—green, O—dark blue, Na—bright blue, Al—yellow, P—pink, Cu—red, and Si—purple).

The SEM micrographs revealed significant changes in the morphology of examined alga. Figures 1, 2, and 3(a) present the morphological differences between natural biomass and biomasses after accumulation.* Vaucheria sessilis* enriched with Zn(II) ions exhibited medium disintegration of a meshwork structure. The dysfunction of typical biomass morphology was observed in* Vaucheria sessilis* enriched with Cu(II) ions. Scanning microscope observations indicated a loss of filaments thickness; additionally a high occurrence of reproductive structures was noticed. Moreover, the biomass enriched with Zn(II) and Cu(II) ions revealed visible deformation of the cell wall.

Additionally, the chemical distribution analysis of all investigated elements confirmed that the bioaccumulation of metal ions took place inside the cell. The distribution of Cu(II) ions on the surface of the biomass was very dense and regularly arranged; however, cross sections revealed heterogeneous locations. Analysis of Zn(II) ions distribution showed a slide aggregation of the mentioned element on the surface of the biomass, whereas a highly aggregated arrangement on the cross section was visible.

Table 2 presents atomic concentration of the elements (%) on the surface and in the cross section of macroalga* Vaucheria sessilis* after bioaccumulation. The changes in atomic concentration on the surface of the biomass after bioaccumulation (b) concerned mainly the increase of carbon concentration (except of MA-Cu) and decrease of oxygen (except of MA-Cu), sulphur, calcium, and sodium (except of MA-Cu). These results stay in agreement with data obtained by ICP-OES. Decrease of the content of Ca(II) and Na(I) ions on the surface of the biomass (increase in the solution) was due to ion exchange with metal ions from the solution—Zn(II) and Cu(II). Atomic concentration of carbon in the cross section of MA-Cu and MA-Zn was lower than in the natural biomass. A different observation concerned the atomic concentration of oxygen. It was also noticed that atomic concentration of sulphur and calcium decreased and sodium increased.

## 4. Conclusions

*Vaucheria sessilis *is a widespread alga but has not been thoroughly studied yet. The performed experiments showed that it can act as a good bioaccumulator of metal ions. Higher bioaccumulation was more observed for Zn(II) than for Cu(II) ions.

The combination of two advanced methods ICP-OES and SEM-EDX allowed to characterize the process of bioaccumulation of metal ions by* Vaucheria sessilis. *The scanning electron microscopy technique allowed us to understand the interaction between metal ions and the biomass. In the case of the surface (biosorption), the elements revealed aggregate distribution, in contrast to the cross section (bioaccumulation) where even arrangements were noticed. The SEM analysis also revealed significant changes in the morphology of the investigated algae. ICP-OES analysis of the solution before and after bioaccumulation showed that metal ions from aqueous solution were bound to the biomass. Bioaccumulation properties of algae may be used in the industry, for example, in the production of natural feed supplements. Additionally, the possibility of algae enrichment in chosen elements may find an application in production of natural fertilizers. The enriched biomass can serve as a rich source of highly bioavailable and nontoxic forms of microelements both for animals as well as for plants.

## Figures and Tables

**Table 1:** The concentration of elements in the solution before and after bioaccumulation of Zn(II) and Cu(II) ions by *Vaucheria sessilis*.

Element	Concentration (mg L^−1^) of elements in the solution:			
	Before	After	Before	After
	Zn(II) ions		Cu(II) ions	
	Bioaccumulation∗ by *Vaucheria sessilis *			
Co	0.0226 ± 0.0056	0.0375 ± 0.0094	<LLD	0.00500 ± 0.00125
Cu	0.0256 ± 0.0064	0.0353 ± 0.0088	**14.0 ± 2.1**	**4.35 ± 0.65**
Fe	0.836 ± 0.125	0.253 ± 0.038	0.833 ± 0.125	0.452 ± 0.068
Mn	0.0551 ± 0.0138	0.0325 ± 0.0081	0.0423 ± 0.0105	0.0937 ± 0.0234
Zn	**15.6 ± 2.34**	**1.81 ± 0.27**	0.099 ± 0.025	0.381 ± 0.057
Ca	2.71 ± 0.41	5.87 ± 0.88	2.14 ± 0.32	6.72 ± 1.01
Na	3.32 ± 0.50	4.18 ± 0.63	6.62 ± 0.99	7.75 ± 1.16
Ba	0.0151 ± 0.0038	0.0333 ± 0.0083	0.00895 ± 0.00224	0.0696 ± 0.0174
K	90.0 ± 13.5	96.5 ± 14.5	88.4 ± 13.3	97.1 ± 14.6
Al	0.124 ± 0.019	<LLD	0.105 ± 0.016	0.0762 ± 0.0190
Mg	2.11 ± 0.32	3.16 ± 0.47	2.11 ± 0.32	4.20 ± 0.63

<LLD: below low limit of detection (Co < 0.0009 mg L^−1^; Al < 0.0234 mg L^−1^).

∗ *C*_0_ 12.5 mg L^−1^; pH of the initial solution 7.0; *C*_*S*_ 0.14 g L^−1^; *T* 23°C, bioaccumulation time: 2 weeks.

**Table 2:** Atomic concentration of the elements (%) on the surface (b) and in the cross section (b′) of macroalga *Vaucheria sessilis *after bioaccumulation.

Element	Atomic concentration of the elements (%)					
	b	b′	b	b′	b	b′
	MA-natural		MA-Cu		MA-Zn	
C	48.2	37.5	42.8	36.3	54.5	37.1
Cl	<LLD	<LLD	<LLD	<LLD	<LLD	<LLD
O	48.2	55.2	52.8	59.4	42.6	59.1
P	0.81	0.96	1.24	0.99	0.92	0.69
S	0.84	0.92	0.51	0.33	0.56	0.37
Co	<LLD	<LLD	<LLD	<LLD	<LLD	<LLD
Cu	<LLD	<LLD	1.23	1.39	<LLD	<LLD
Fe	0.17	0.61	0.18	0.33	0.09	0.6
Mn	<LLD	<LLD	<LLD	<LLD	<LLD	<LLD
Zn	<LLD	<LLD	<LLD	<LLD	0.01	1.11
Ca	0.44	0.71	*0.25 *	*0.23 *	*0.29 *	*0.2 *
Na	0.57	0.67	0.92	0.89	0.21	0.75
Al	0.25	2.46	0.01	0.11	0.47	0

<LLD: below detection limit, *Italics*: below or above the detection limit.

Upper and lower limit of detection (%): Cl (0.001–2.621); Co (0.076–6.924); Cu (0.083–8.040); Mn (0.063–5.894); Zn (0.001–8.630).
